# Skin Hyperpigmentation as the Presenting Symptom of Subacute Combined Degeneration of the Spinal Cord

**DOI:** 10.1155/2017/7140908

**Published:** 2017-11-22

**Authors:** Abdulhameed Alhazmi, Abdulrahman Almalki, Safieeldin Ghazala

**Affiliations:** ^1^Department of Internal Medicine, Prince Sultan Military Medical City, Riyadh, Saudi Arabia; ^2^Department of Neurology, King Abdulaziz Hospital, Jeddah, Saudi Arabia; ^3^Department of Radiology, Mostaqbal Hospital, Jeddah, Saudi Arabia

## Abstract

Vitamin B12 deficiency results in hematological, neurological, and rarely dermatological complications. Subacute combined degeneration of the cord is one of the neurological complications, and usually the presenting symptom is paresthesia. Herein, we report a case of a 46-year-old man with subacute combined degeneration presenting with knuckle hyperpigmentation.

## 1. Introduction

Subacute combined degeneration (SCD) is one of the neurological sequels to vitamin B12 deficiency. It is characterized by degenerative process involving posterior and lateral columns of the spinal cord and presents usually with paresthesia in the toes, fingers, or both [1].

Hyperpigmentation represents the most common associated dermatological finding in vitamin B12 deficiency, although its presence in SCD has only been reported in a very few cases in the literature [1–3].

## 2. Case Report

**Figure 1:**
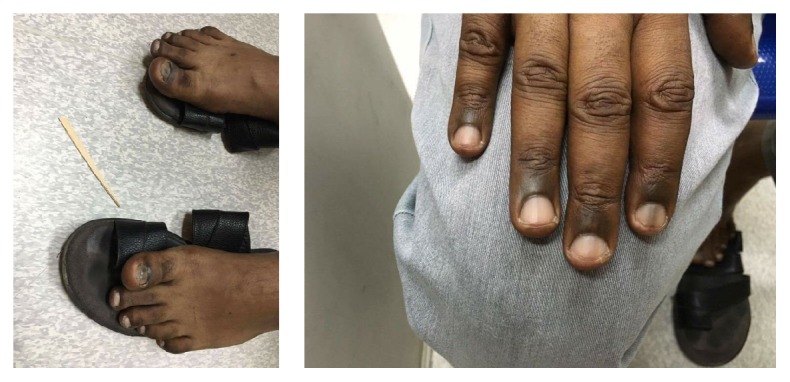
Marked hyperpigmentation over the knuckle pads (interphalangeal joints) and (periungual areas) of the hands and feet.

A 46-year-old gentleman from Sudan without any chronic medical illness or drug use presented with fatigue and hyperpigmentation on the dorsal aspects of the hands and feet. He visited dermatologist and endocrinologist who ruled out common conditions such as Addison's disease and hypothyroidism. Four to six weeks later, the patient developed weakness, numbness, and tingling sensation in both hands and feet. Examination revealed a brownish discoloration on the dorsal aspects of hands and feet and at the distal interphalangeal joints and proximal and distal metatarsal joints. It was not itchy, associated with leukonychia and melanonychia on hands and toes' nails (Figure 1). Neither vitiligo nor stomatitis was observed. On neurological examination, hyporeflexia in both upper and lower limbs with positive planter reflex was observed with normal power. Vibration and proprioception were reduced in elbow and wrist, with more significant reduction in knees and ankles.

**Figure 2:**
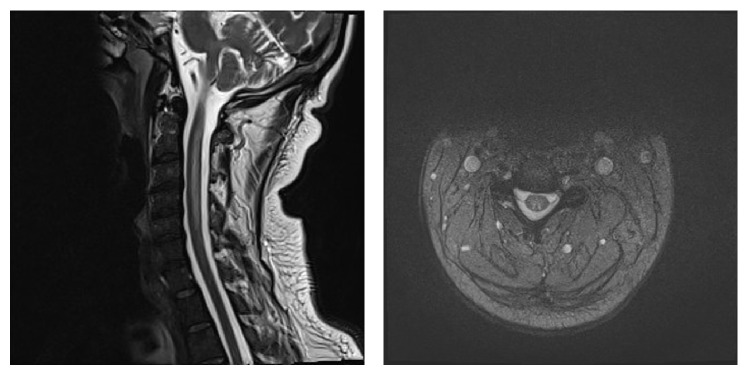
T2 weighted MRI in both axial and sagittal views of the spine shows long segment of abnormal high signal involving the posterior aspect of the cervical cord extending from C1 to C5.

His laboratory results are shown in Table 1. The peripheral blood smear showed pancytopenia with RBCs anisocytosis, occasional schistocytes, and no blasts. The bone marrow was hypercellular and was supporting the diagnosis of megaloblastic anemia. T2 weighted MRI in both axial and sagittal views of the spine shows long segment of abnormal high signal involving the posterior aspect of the cervical cord extending from C1 to C5 (Figure 2). A diagnosis of pernicious anemia as a cause of the vitamin B12 deficiency was made after a positive anti-intrinsic factor antibodies test.

The patient was given intramuscular injections of vitamin B12 1000 mcg three times a week for one week, followed by one injection weekly for a month, followed by one injection every month for the rest of life

After 2-month review, after six weeks of the treatment, the hyperpigmentation was resolved. The neurological symptoms are getting better, although they have not subsided entirely.

## 3. Discussion

Vitamin B12 is a water-soluble vitamin that plays an important role in DNA synthesis, resulting in multisystem complications when the level in the serum is deficient. The neurological complications of vitamin B12 deficiency include mental changes, peripheral neuropathy, and SCD [4]. The most common cause of SCD is pernicious anemia; it was the cause in this case as well. It is defined by the presence of antibodies against the parietal cells of the stomach which produce intrinsic factor, resulting in insufficient absorption of vitamin B12.

Hyperpigmentation is a known dermatological manifestation of vitamin B12 deficiency. The mechanism is due to the increased melanin levels in the epidermis basal layer. The presence of huge numbers of melanosomes in melanocytes and circling keratinocytes was observed in an electronic microscope. Two cases of vitamin B12 deficiency which presented initially with darkening of palms and soles were reported in the literature [5, 6]. However, the presence of hyperpigmentation in a diagnosed case of SCD is rarely mentioned, and it was in a pediatric age group [1, 2]. The causes of SCD were due to dietary lacking and unclear etiology, respectively.

The dorsa of the hand and foot are the most common sites of the hyperpigmentation in a patient with B12 deficiency. Whether the development of the hyperpigmentation is responding to a certain decrease level of vitamin B12 in the serum or not still remains a question. In SCD-associated hyperpigmentation, the level varies between 60 and 121 pg/mL. However, there was one case in India that developed knuckle hyperpigmentation and the level of vitamin B12 was 31.6 pg/mL without presence of SCD [7]. The onset of signs and symptoms of SCD after the hyperpigmentation is also not crystal clear. It is estimated to be six weeks in our case, but it was not defined in the other cases. Despite the unclear bond between vitamin B12 levels and the onset of hyperpigmentation in SCD, the resolution period of the hyperpigmentation after the treatment tends to be from 6 weeks to 12 weeks, except for the case reported by Kumar and Sharma which took 6 months [7]. That can mean it depend more on the patient's response to the treatment.

The delay in the diagnosis and treatment will cause SCD symptoms to progress to the extent that they can be irreversible. It is important to look for SCD in a patient who presents with hyperpigmentation regardless of the presence of the neurological symptoms, because they can manifest late.

## Figures and Tables

**Table 1:** Laboratory findings.

Investigation	At presentation	6 weeks after treatment	Reference range
Hemoglobin (gm/dL)	10.3	13.4	12.5–17.5 gm/dL
Mean corpuscular volume (MCV) (fL)	112	94.6	78–100 fL
Serum vitamin B12 (pg/mL)	83	>2000	200–1200 pg/mL
Serum folic acid (ng/mL)	>20	13.75	4.6–18.7 ng/mL
Methylmalonic acid (MMA) (nmol/l)	54.800	—	87–318 nmol/l
Anti-intrinsic factor antibodies	Positive		
Antinuclear antibody (ANA)	Negative		
